# Giant Hydatid Cysts of the Lung and Liver

**DOI:** 10.1590/0037-8682-0492-2019

**Published:** 2020-03-16

**Authors:** Recep Tekin, Rojbin Ceylan Tekin, Alper Avcı

**Affiliations:** 1 Dicle University, Faculty of Medicine, Department of Infectious Diseases and Clinical Microbiology, Diyarbakir, Turkey.; 2 Genesis Hospital, Department of Radiology, Diyarbakir, Turkey.; 3 Çukurova University, Faculty of Medicine, Department of Thoracic Surgery, Adana, Turkey.

A 26-year-old girl from a region endemic for hydatid disease was admitted with a 3-month history of progressive dyspnea and abdominal pain. Physical examination of the abdomen showed tenderness in the right upper quadrant. Eosinophilia was detected in a peripheral blood sample, and serum IgG against *Echinococcus granulosus* was positive (titer 1/160) using the immunofluorescence assay test. A thoraco-abdominal contrast enhanced computed tomography showed one cyst (15 × 14 cm) (Figure A) in the right lobe of the liver and another cyst (14 × 11 cm) in the right lobe of the lung (Figure B). The patient underwent enucleation of the cyst with capitonnage via a one-stage posterolateral thoracotomy. Liver cystectomy was also performed. Histopathological examination of the cysts confirmed the diagnosis of hydatid disease. After an uneventful postoperative period, the patient was discharged on the 20^th^postoperative day. Hydatid Cyst should be considered in the differential diagnosis of patients presenting thoracic cysts, particularly in those who live in endemic areas. The most common sites of lodgment of*E. granulosus*are the liver and lung^1^. It is very rare for both pulmonary hydatid cyst and liver hydatid cyst to exist in large sizes^2^ ^,^ ^3^. The diagnosis of hydatid disease should be considered in patients with giant cysts in the lungs and liver. 

**FIGURE A:**
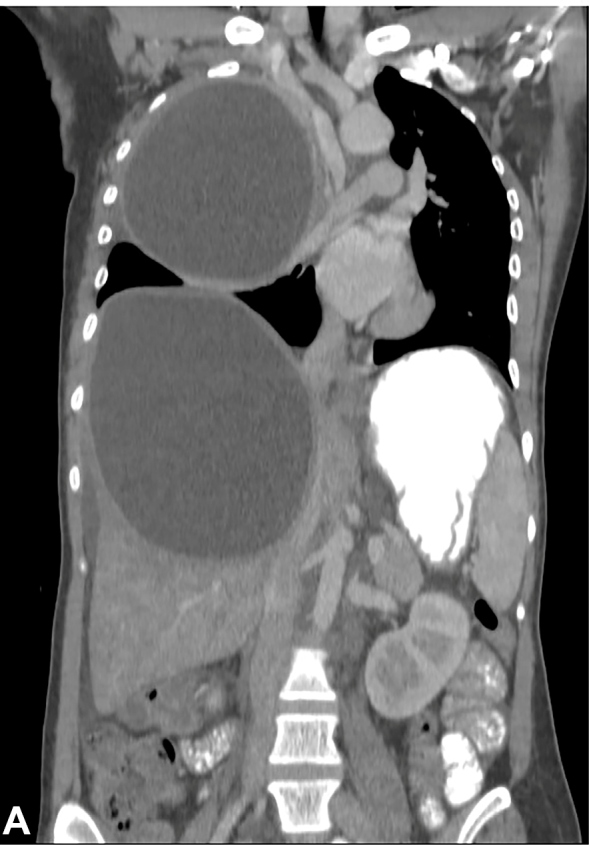
Computed tomographic scan of the abdomen showing one cyst (15 × 14 cm) in the right lobe of the liver.

**FIGURE B:**
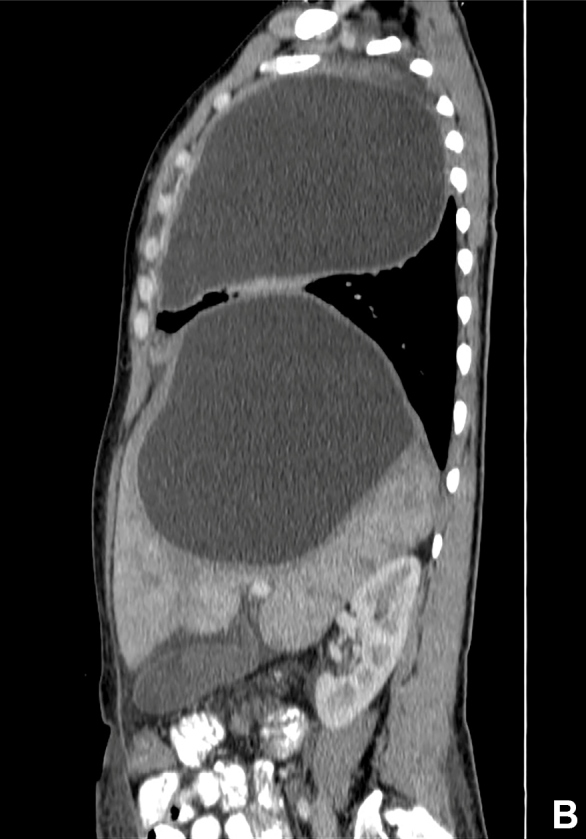
Computed tomographic scan of the chest showing one cyst (14 × 11 cm) in the right lobe of the lung.
